# Plasma Lysosphingomyelin Demonstrates Great Potential as a Diagnostic Biomarker for Niemann-Pick Disease Type C in a Retrospective Study

**DOI:** 10.1371/journal.pone.0114669

**Published:** 2014-12-05

**Authors:** Richard W. D. Welford, Marco Garzotti, Charles Marques Lourenço, Eugen Mengel, Thorsten Marquardt, Janine Reunert, Yasmina Amraoui, Stefan A. Kolb, Olivier Morand, Peter Groenen

**Affiliations:** 1 Actelion Pharmaceuticals Ltd, Allschwil, Switzerland; 2 Hospital das Clínicas de Ribeirão Preto, São Paulo, Brazil; 3 Department of Lysosomal Storage Disorder, Villa Metabolica, Center for Paediatric and Adolescent Medicine, University Medical Center of the Johannes Gutenberg University Mainz, Mainz, Germany; 4 Klinik für Kinder- und Jugendmedizin, Münster, Germany; Medical University of South Carolina, United States of America

## Abstract

Niemann-Pick disease type C (NP-C) is a devastating, neurovisceral lysosomal storage disorder which is characterised by variable manifestation of visceral signs, progressive neuropsychiatric deterioration and premature death, caused by mutations in the *NPC1* and *NPC2* genes. Due to the complexity of diagnosis and the availability of an approved therapy in the EU, improved detection of NP-C may have a huge impact on future disease management. At the cellular level dysfunction or deficiency of either the NPC1 or NPC2 protein leads to a complex intracellular endosomal/lysosomal trafficking defect, and organ specific patterns of sphingolipid accumulation. Lysosphingolipids have been shown to be excellent biomarkers of sphingolipidosis in several enzyme deficient lysosomal storage disorders. Additionally, in a recent study the lysosphingolipids, lysosphingomyelin (SPC) and glucosylsphingosine (GlcSph), appeared to be elevated in the plasma of three adult NP-C patients. In order to investigate the clinical utility of SPC and GlcSph as diagnostic markers, an in-depth fit for purpose biomarker assay validation for measurement of these biomarkers in plasma by liquid chromatography-tandem mass spectrometry was performed. Plasma SPC and GlcSph are stable and can be measured accurately, precisely and reproducibly. In a retrospective analysis of 57 NP-C patients and 70 control subjects, median plasma SPC and GlcSph were significantly elevated in NP-C by 2.8-fold and 1.4-fold respectively. For miglustat-naïve NP-C patients, aged 2–50 years, the area under the ROC curve was 0.999 for SPC and 0.776 for GlcSph. Plasma GlcSph did not correlate with SPC levels in NP-C patients. The data indicate excellent potential for the use of lysosphingomyelin in NP-C diagnosis, where it could be used to identify NP-C patients for confirmatory genetic testing.

## Introduction

Niemann-Pick disease type C (NP-C) is caused by mutations in either the *NPC1* or the *NPC2* gene, it is a rare neurovisceral lysosomal storage disorder (LSD) which leads to progressive neuropsychiatric deterioration and in the majority of cases, premature death [1]. The visceral, neurological and psychiatric manifestations observed in NP-C patients are heterogeneous in their presentation and are shared with other disorders complicating diagnosis [2]. The most recent analysis found a significant discrepancy between average on-set of neurological symptoms (10.9±9.8 years) and diagnosis (15.0±12.2 years) [3]. Additionally, there is increasing evidence from epidemiological studies that there may be a pool of patients who only become symptomatic later in-life and consequently remain undiagnosed [2], [4].

Recent efforts have aimed to score the symptomatology of NP-C using a disease-specific Suspicion Index [5], as well as disease scales [6], [7]. Tools like the NP-C Suspicion Index should help channel symptomatic patients towards expert medical centers for appropriate clinical evaluation, and genetic and biochemical diagnostic tests. The existence of an approved therapy for NP-C in around 40 countries (with the notable exception of the United States) and current efforts by the National Institutes of Health to explore new therapies serve to underline the need for improved methods of diagnosing this devastating disease.

Until recently the diagnosis of NP-C was based primarily on the filipin test, in which skin fibroblast cultures are stained for lysosomal cholesterol accumulation [1]. The filipin test is technically challenging, invasive and costly due to the requirements of a skin biopsy and fibroblast culture in a specialized laboratory. The test can lead to non-conclusive results, particularly for adult and juvenile patients. NPC1 and NPC2 gene sequencing would appear in the current age to offer a less fallible means of testing, however it failed to identify 14% patients in a recent study [8] and many physicians still favour biochemical confirmation through the filipin test. Recently, the levels of two oxysterol molecules in plasma have been shown to have excellent specificity and sensitivity in differentiating NP-C patients from controls [9], [10]. Although oxysterols perform very well, it is likely that they will not be 100% specific and sensitive in larger cohorts and the assay requires a complex chemical derivatisation procedure that can be difficult to implement in a sufficiently robust method. Thus new methods probing other aspects of NP-C offer the possibility to improve overall diagnostic accuracy, while also making-up for technical shortcomings in existing methods.

It has recently become apparent that plasma levels of the N-deacetylated, lyso forms of sphingolipids are increased in patients with LSDs in which the activity of enzymes involved in complex sphingolipid degradation is deficient such as Fabry [11], Gaucher [12], GM2 gangliosidoses [13], Krabbe [14] and Niemann-Pick disease type B [15]. The increase in plasma lysosphingolipids can be more than an order of magnitude greater than that for the equivalent sphingolipid, making the lysosphingolipids potentially powerful biomarkers for both diagnosis and monitoring of treatment effects in their respective LSDs.

**Figure 1:**
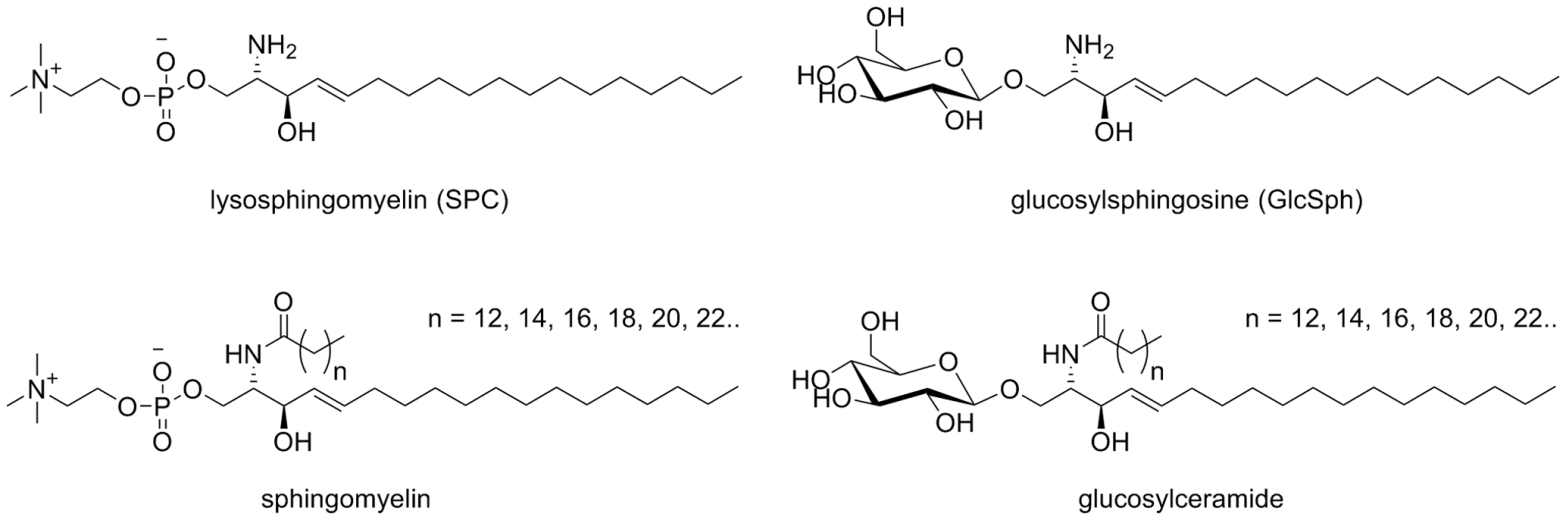
Chemical structures of sphingolipids. The lysosphingolipids SPC and GlcSph measured in plasma for this study, along with their N-acetylated counterparts, sphingomyelin and the monohexosylceramide glucosylceramide which are known to accumulate in the organs of NP-C patients.

NP-C differs from the sphingolipid LSDs in that the aberrant protein is not an enzyme involved in breakdown of sphingolipids, but in 95% of cases a membrane protein (NPC1) whose disruption leads to a complex intracellular endosomal/lysosomal trafficking defect [1]. As a consequence of this defect along with unesterified cholesterol, a large number of different complex sphingolipids accumulate with differing organ specificity in both NP-C patients and animal models [1], [16], [17], [18]. This led to the hypothesis that a panel of lysosphingolipids may be useful as biomarkers in NP-C. This hypothesis was previously tested in the ZOOM study, a multicentre genetic screening study of adult patients with neurological and psychiatric symptoms [4]. The three adult NP-C patients identified in the ZOOM study appeared to have elevated plasma levels of both lysosphingomyelin (SPC) and hexosylsphingosine (GlcSph) (Figure 1) when compared to NP-C negative patients.

Data from an in-depth technical assay validation for measurement of plasma SPC and GlcSph are reported here. The two lysosphingolipids were measured in the plasma of 57 NP-C patients in a retrospective study and SPC is shown to have potential diagnostic utility.

## Materials and Methods

### Materials

SPC (CAS 1670-26-4) and C17-SPC (CAS 118540-32-2) were from Avanti Polar Lipids, Inc (Alabaster, AL, USA). GlcSph (CAS 52050-17-6), galactosylsphingosine (GalSph) (CAS 2238-90-6) and 1-b-D-D-glucosylsphingosine (D-GlcSph; CAS 124649-06-5) were from Matreya LLC (Pleasant Gap, PA, USA). Pooled EDTA-plasma used in assay validation was from United States Biologicals (Salem, MA, USA). Plasma used for assessing matrix effect and EDTA-blood stability experiments was from Actelion Pharmaceuticals Ltd, Immunology Biology Department, Allschwil, Switzerland. Plasma samples for the control group came from Tissue Solutions (Glasgow, UK), The Geneticist (Glendale, Co, USA) and PrecisionMed Inc (Solana Beach, Ca, USA). Solvents were from Sigma Aldrich (Buchs, Switzerland) and were of HPLC grade.

### Assay validation

Before commencing assay validation a full assay validation plan was written according to FDA and EMA guidelines for bioanalytical methods to aid the experimental design [19], [20] and a set of acceptance criteria was designed. To determine the accuracy of the method with the quality control (QC) samples an adaption of the method for LC-MS/MS biomarker validation described by Houghton et al was used [21]. The nominal concentration of QC2 was defined as the average measured value for the three validation batches. The nominal concentrations of QC3 and QC4 were the nominal concentration of QC2 plus the respective spiking concentrations. The standard validation batch used for determination of precision and accuracy consisted of duplicate calibration curves, duplicate blank sample (unspiked surrogate matrix) and six replicates of each QC sample.

### Preparation of calibration solutions and quality controls

Working solutions of GlcSph and SPC 100-fold above the final concentrations were prepared in CHCl3/MeOH 2∶1. For preparation of the calibration (CAL) and quality control (QC) solutions, surrogate matrix (EDTA-plasma pool, 10-fold diluted with PBS buffer) and EDTA-plasma pool respectively were fortified with the working solutions using a ratio of 99/1 (v/v). Following preparation of the CAL and QC solutions 130 µl aliquots were frozen at −20°C. The nine CALs covered the range of 5–500 nM and 0.5–50 nM for SPC and GlcSph respectively.

### Sample preparation

For each sample (CAL, QC or study sample), 100 µL of sample was added to 900 µL of solution A (25% MeOH +0.1% H_3_PO_4_) containing the internal standards (ISTDs) (1.5 nM of C17-SPC and 0.5 nM of D-GlcSph). The tubes were placed in a 96-well plate (96WP) format rack and mixed at 1000 rpm for 10 min at 37°C. The samples were transferred (900 µL) into the wells of the solid phase extraction (SPE) 96-well plate (Waters OASIS HLB 96Well Plate 30 mg) previously primed with: 1 mL hexane, 1 ml methanol, 2×1 mL solution A). The sample was loaded on the SPE matrix by vacuum, the SPE matrix was washed twice with 1 mL of solution A and 1 mL of solution B (25% MeOH). The lysosphingolipids were eluted with 1.2 mL of solution C (100% MeOH +0.01% v/v NH_4_OH) (three additions of 400 µL). The solution eluted from the SPE matrix was dried under a stream of heated nitrogen (60°C). For LC–MS/MS analysis, 60 µL of solution D (90% MeOH +0.1% v/v HCOOH) was added to each tube. The samples in the 96WP rack were vortexed for 10 min at room temperature, sonicated for 10 min in an ultrasound bath and then centrifuged for 5 min at 2000 g. The supernatant was transferred to a new 96WP and 5 µL were injected into the LC-MS/MS system.

### LC-MS/MS

Experiments were performed with a ABSciex QTRAP6500 equipped with a Dionex UltiMate 3000 HPLC unless otherwise noted. The instrument was run in positive ion electrospray mode with the following source parameters curtain gas (30); collision gas (high) Q1 and Q3 resolution (unit); ion spray voltage (5500); temperature (550); gas1 (55) and gas2 (50). The following transitions were used for quantification (Q1/Q3); SPC (465.3/184); C17-SPC (451.3/184); GlcSph (462.3/282.1) and D-GlcSph (460.3/280.1). For secondary qualitative assessment for interferences the following transitions were used SPC (465.3/125); C17-SPC (451.3/125); GlcSph (462.3/264.1) and D-GlcSph (460.3/262.1). The dwell times for individual quantitative and qualifier transitions were 40 and 10 ms respectively. Other parameters (EP, DP, CE, and CXP) were optimized per transition using standard procedures.

The HPLC autosampler was maintained at 15.0°C with an autosampler wash of water/methanol (25/75, v/v). The column oven temperature was 55.0°C. Buffer A was 100% water +0.1% v/v HCOOH. Buffer B was 50∶50 acetone∶acetonitrile with 0.1% v/v HCOOH. The HPLC column and conditions were similar to those described [22]. An ACE 3 C8, 50×2.1 mm ID (ACE-112-0502) with a guard-column ACE 3 C8, 2.1 mm (ACE-112-0102GD) at a flow rate of 0.9 mL/min was used. A gradient was run from 10 to 66% buffer B over the first 4 min, followed by cleaning with 100% buffer B for 1minute and 0.5 min of re-equilibration with 10% buffer B.

### Matrix effect

Plasma samples from six individual donors were extracted as described above and then reconstituted in a 90% methanol solution containing the internal standards (ISTDs) (30 nM C17-SPC and 10 nM D-GlcSph) and the two analytes SPC and GlcSph at two concentration levels (20 nM SPC, 2 nM GlcSph as low level and 400 nM SPC, 40 nM GlcSph as high level) in four replicates. Matrix factors (MF) and ISTD normalized MFs were calculated using standard methods.

### EDTA-blood stability experiment

Fresh EDTA-blood was collected and divided into 7×600 µL aliquots. An aliquot was immediately centrifuged for 10minutes at 20°C and 2000 g in order to prepare EDTA-plasma and frozen on dry ice. The remaining 6 aliquots were stored at room temperature and plasma samples were prepared following the same procedure after 30 min, 1 h, 2 h, 3 h, 4 h and 5 h.

### Incurred sample reanalysis

Variability was calculated as defined in [23], using the equation. Variability  = 100*(Repeat-original)/mean.

### Acceptance criteria for sample-sets

All CALs were to be run in duplicate and QCs in duplicate or quadruplicate. A sample-set was to be considered valid if 66% of the QCs were within 15% of the validation defined concentration (20% for QC2), including at least 50% at each level. At least two-thirds of the CAL samples had to be within ±15% of their respective nominal values. A tolerance of ±20% was allowed for CAL1. If neither of the two CAL1 samples reached the tolerance of ±20%, the batch was to be repeated. If one analyte failed to meet the acceptance criteria, the batch was to be repeated, but the data for the accepted analyte from the first run were to be used.

### Glucosyl- and galactosylsphingosine separation

The samples were prepared as per the standard method except 200 µL plasma was loaded on the SPE cartridge. The chromatographic method consisted of an isocratic gradient of acetonitrile∶water∶methanol 86∶7∶7 containing 315 mg/L of ammonium formate and 0.1% v/v formic acid on an Atlantis HILIC Silica 5 µm, 150×2.1 mm column (Waters Part No 186002016).

### Cholestan-3β,5α,6β-triol measurement

Cholestan-3β,5α,6β-triol was measured using a GCMS method adapted from that in Porter et al. [10] (Reunert et al. manuscript in preparation).

LC-MS/MS data was processed with MultiQuant 2.1 (ABSciex) with some further statistical assessment in Excel (Microsoft). Column statistics, Kruksal-Wallis (with Dunn's multiple comparison test), Mann Whitney, Pearson correlations and receiver operating characteristic (ROC) analysis were performed using Graphpad Prism 6.0.

### NP-C patients and control subjects

All NP-C patients and controls had given written consent to the use of their sample for biomarker measurements. The consent form had been approved by the relevant local committees (Ethic Comitee from Hospital das Clinicas de Ribeirao Preto (HCRP), University of Sao Paulo and Landesärztekammer Rheinland-Pfalz). NP-C patients had been previously diagnosed as NP-C based on gene sequencing (46%), filipin staining (7%), or both (47%). Age and sex demographics on the cohorts are given in table 1. The control group comprised 70 samples from five different sources. Thirty five of the control samples were purchased from three different commercial suppliers of biosamples. The remaining samples came from the same centers as the NP-C patients and a number had similar symptoms.

**Table 1:** Demographics of the cohorts.

Group	Control	NP-C
Number of values	70	57
Male (%)	44.3	42.1
Female (%)	55.7	57.9
Age (yrs) minimum	1	2
Age (yrs) 25% percentile	9	11
Median age (yrs)	16	15
Age (yrs) 75% percentile	25	22
Age (yrs) maximum	69	72

## Results

Plasma SPC and GlcSph were measured using LC-MS/MS and the elution profile of the analytes and internal standards (ISTD) can be seen in Figure S1 in File S1.

### Bioanalytical precision and accuracy

The descriptive statistics of the plasma quality control (QC) samples for the three main validation batches are presented in Table 2. The QCs gave good precision across the three validation batches. The accuracy of the assay for measurement of the spiked plasma samples QC3 and QC4 was also acceptable when the high precision and intended use of the method are taken into account [24].

**Table 2:** Precision and accuracy of the QC plasma samples.

Batch	Value	SPC QC2	SPC QC3	SPC QC4	GlcSph QC2	GlcSph QC3	GlcSph QC4
	nominal concentration (nM)	6.4	106.4	366.4	0.7	10.7	36.7
batch 1	CV [%]	5.5	2.4	1.9	4.0	5.1	4.0
	accuracy [%]	108.0	84.5	97.4	118.5	108.9	113.7
batch 2	CV [%]	2.5	2.0	6.1	7.9	5.0	5.8
	accuracy [%]	101.6	86.2	94.6	92.4	105.9	115.1
batch 3	CV [%]	5.0	4.7	3.6	8.8	3.6	7.1
	accuracy [%]	90.5	84.2	95.0	89.0	111.7	117.9
all	mean concentration (nM)	6.4	90.4	350.5	0.7	11.6	42.4
	mean intra-plate CV [%]	4.3	3.1	3.9	6.9	4.6	5.6
	inter-plate CV [%]	8.6	3.2	4.2	15.0	4.8	5.7
	inter-plate accuracy [%]	100.0	85.0	95.7	100.0	108.8	115.6

Shown are the precision and accuracy for each analyte at 3 levels in 3 batches (N = 6 per batch) and the inter batch statistics. The nominal concentration of QC2 was defined as the average measured value for the three validation batches. The nominal concentrations of QC3 and QC4 were the nominal concentration of QC2 + the respective spiking concentrations. The precision (coefficient of variation CV) and accuracy (%, where 100% is the actual value) are given for each of the individual batches and for the data-set as a whole.

### Matrix effect

The matrix effect was assessed using EDTA-plasma from 6 different donors and 2 spiking concentrations of the analytes (Table S1 in File S1). In all cases the matrix factor was found to be close to 1 and the CV of the internal standard normalized matrix factor was <10%. This indicates that the matrix effects were negligible and that between the six different donors there is minimal variation in matrix effect.

### Stability experiments

**Figure 2:**
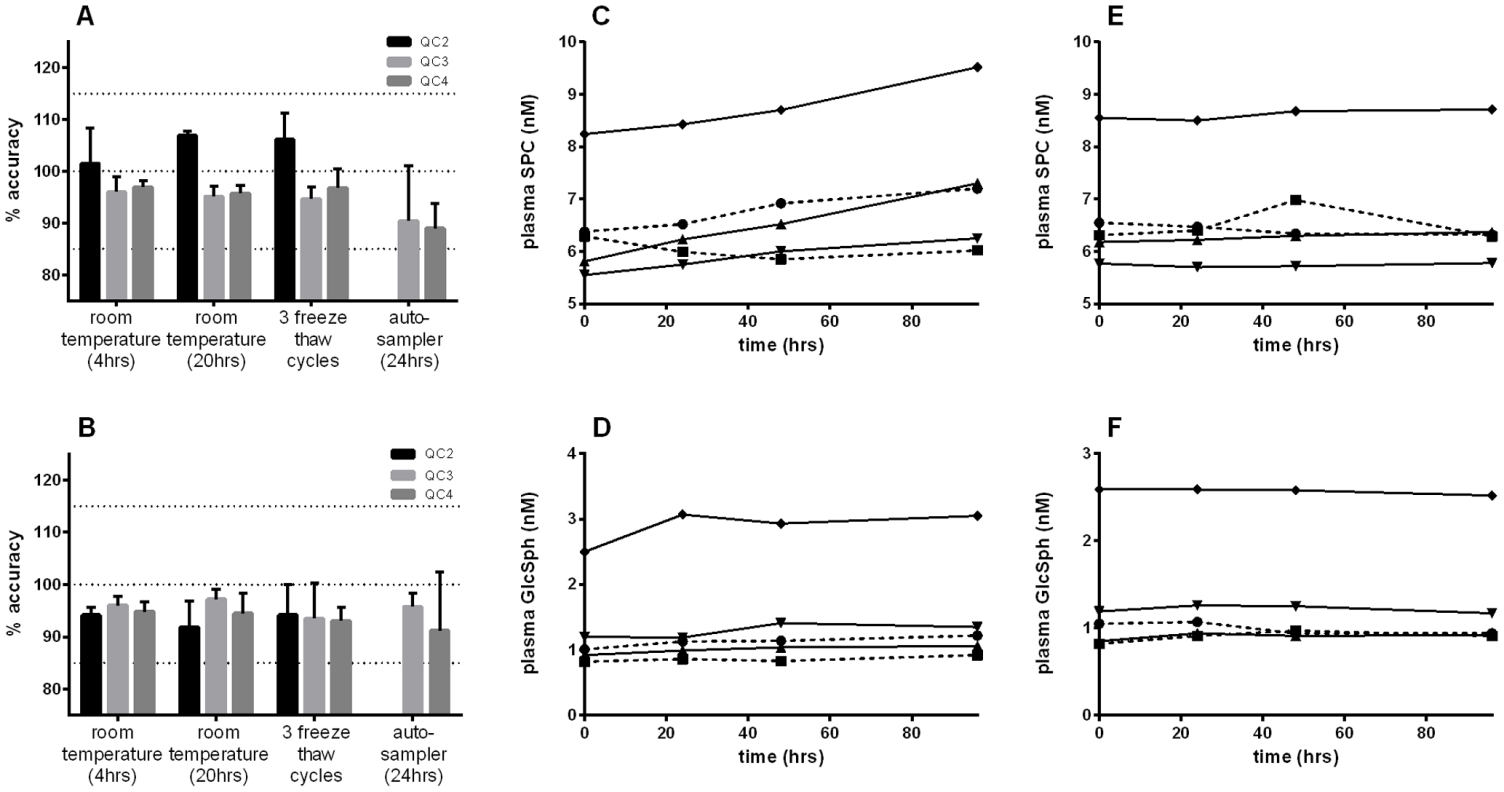
Stability of GlcSph and SPC in plasma. A and B show the stability of the plasma QC samples under different conditions for SPC and GlcSph respectively. Percentage measured compared to the average concentration of the samples determined in the three validation batches is shown. Data are reported +/− standard deviation for 3 replicate measurements. Note the observation of all data being a little under 100% represents a systematic bias from this particular batch; Stability of SPC and GlcSph in EDTA-plasma from five donors incubated at room temperature (C and D) and 4°C (E and F) for up to 96 hours. Each trace represents an individual donor.

Both analytes were found to be stable in the plasma QC samples when stored at room temperature or 4°C for 24 h, after three freeze-thaw cycles and after 24 h in the autosampler post extraction (Figure 2A and 2B).

Data have been collected on the measurements of QC and calibrants (CALs) over a 4-month period. The only noticeable drift has been in QC2 for SPC, with an increase of the measured concentration of about 40% compared to the value determined at the start of the validation when stored at −20°C (Figure S2 in File S1). This observation led us to perform additional experiments to further investigate the analyte stability, de-risk assay performance and, importantly, to examine conditions that may realistically occur in a clinical setting.

Plasma stability was tested in samples from five donors for up to 96 h at both room temperature and 4°C (Figures 2C-F). Both analytes showed good stability after 96 h at room temperature, the levels of SPC and GlcSph had increased by only 13% (p = 0.058) and 17% (p = 0.048) respectively (2-tailed paired t-test). When the plasma was maintained at 4°C after 96 h the analytes were completely stable, with only a negligible increase of 0.76% and 0.25% for SPC and GlcSph respectively. The stability in fresh EDTA-blood at room temperature was also tested in samples from three different donors (Figure S3 in File S1), both analytes were completely stable within the limits of the experiment showing an average increase of only ∼4% during 5 h.

### EDTA-plasma and heparin-plasma

The SPC and GlcSph levels were assessed in plasma samples taken from blood with either heparin or EDTA anticoagulant from 10 donors. A paired t-test indicated there was no difference in going from EDTA- to heparin-plasma with differences of −0.6% (p = 0.77) for SPC and −5.2% (p = 0.26) for GlcSph.

### Robustness

A set of CALs and QCs was run on two different LC-MS/MS systems that were not used during the assay validation. In both cases the acceptance criteria were met for the calibration curves and the concentration of the QC samples (Table S2 in File S1).

### Incurred sample reanalysis

A group of 58 samples coming from four different sites was analyzed twice. The variability was <20% for 74% of samples for both SPC and GlcSph and was <30% for 91% and 84% of samples for SPC and GlcSph respectively. A similar experiment performed with 10 control samples stored at −80°C and 3 months apart gave variability of <20% for 90% (SPC) and 100% (GlcSph) of samples.

### Measurement in NP-C patients

Plasma SPC and GlcSph were measured retrospectively in a cohort of 57 NP-C patients and was compared to a control group comprising of 70 samples.

**Figure 3:**
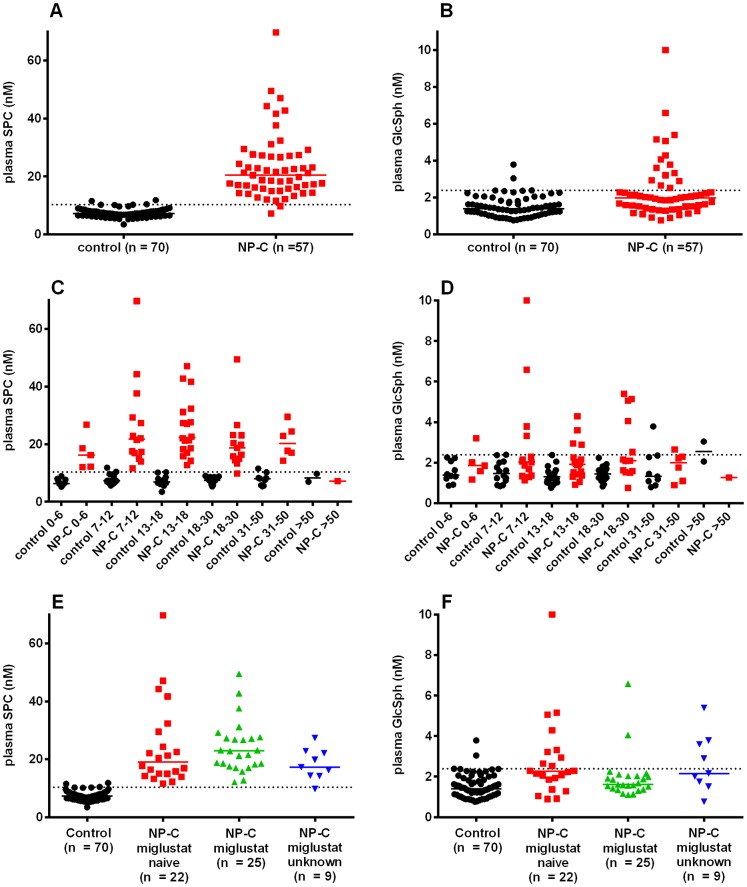
Plasma SPC and GlcSph in NP-C patients and controls. One sample per patient is shown, where multiple samples from one patient were available the first was used. The dotted horizontal line represents the 95% percentile of the entire control group for SPC (10.3 nM) and GlcSph (2.4 nM). The bar is the median. A and B, SPC and GlcSph in the entire cohort; C and D, SPC and GlcSph separated by age (years); E and F, SPC and GlcSph separated by miglustat status (0–50 years). The miglustat treated patients had been on treatment for 2.8±1.4 years (average ± standard deviation), 0.7–6 years (Min-Max).

Median plasma SPC was 2.8-fold higher in NP-C patients than controls, with almost no overlap between the two groups (Figure 3A and Table S3 in File S1). Median plasma GlcSph was 1.4-fold significantly elevated in the NP-C group compared to the control group (Mann-Whitney), although there were a significant number of NP-C patients with GlcSph within the normal range (Figure 3B). When the groups were split based on age, SPC was seen to be elevated independently (Figure 3C), with the exception of the single patient in the >50 years age sub-group. There was also no obvious influence of age on the GlcSph elevation (Figure 3D). The NP-C group in the age range 0–50 years was subsequently split based on treatment with the glucosylceramide synthase inhibitor miglustat (Figure 3E and 3F). SPC was not significantly affected by miglustat treatment (Kruksal-Wallis test). The miglustat-treated NP-C sub-group had lower GlcSph than the miglustat-naïve sub-group (Figure 3F). This comparison in itself did not reach significance (Kruksal-Wallis test with Dunn's multiple comparison test). However, only the miglustat-naïve sub-group had significantly more GlcSph than the controls.

**Figure 4:**
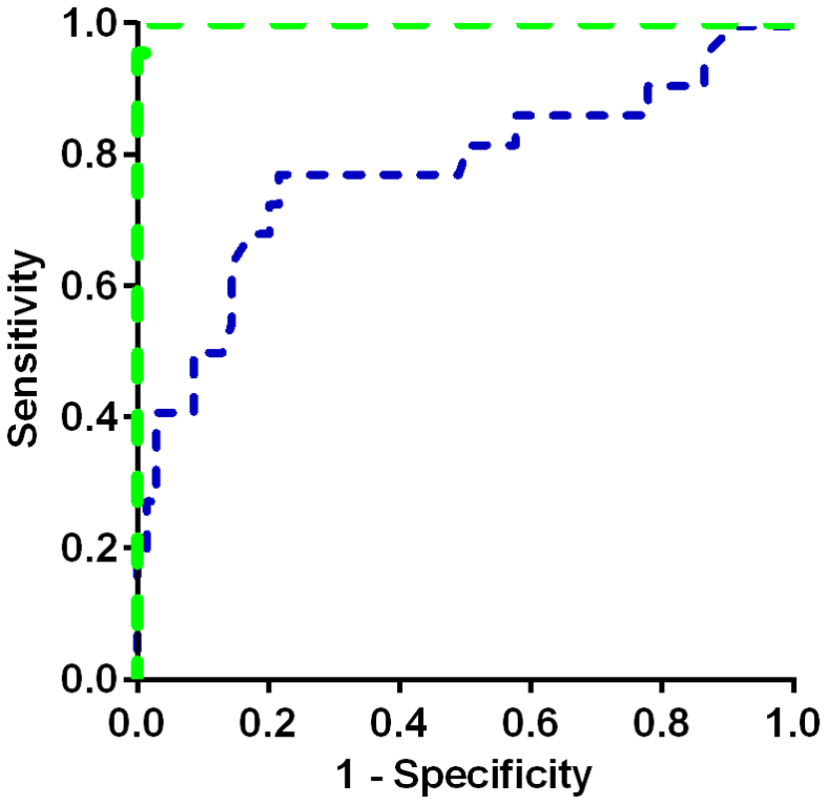
ROC analysis of plasma SPC and GlcSph. SPC (green) and GlcSph (blue) for the age range 0–50 years with miglustat-naïve NP-C patients compared to the control group. The area under the curve (95% CI) was 0.9994 (0.9972 to 1.002) and 0.7764 (0.6479 to 0.9048) for SPC and GlcSph respectively.

A ROC analysis was performed to assess the ability of plasma SPC and GlcSph to separate miglustat-naïve NP-C patients in the age range 0–50 years from controls (Figure 4). SPC and GlcSph gave areas under the curve of 0.9994 and 0.7764 respectively. A cut-off of 11 nM for SPC would provide a sensitivity of 100% and specificity of 97%. Notably the ROC analysis does not in this case determine the true diagnostic sensitivity and specificity because it is not run in a population suspected of having NP-C.

**Figure 5:**
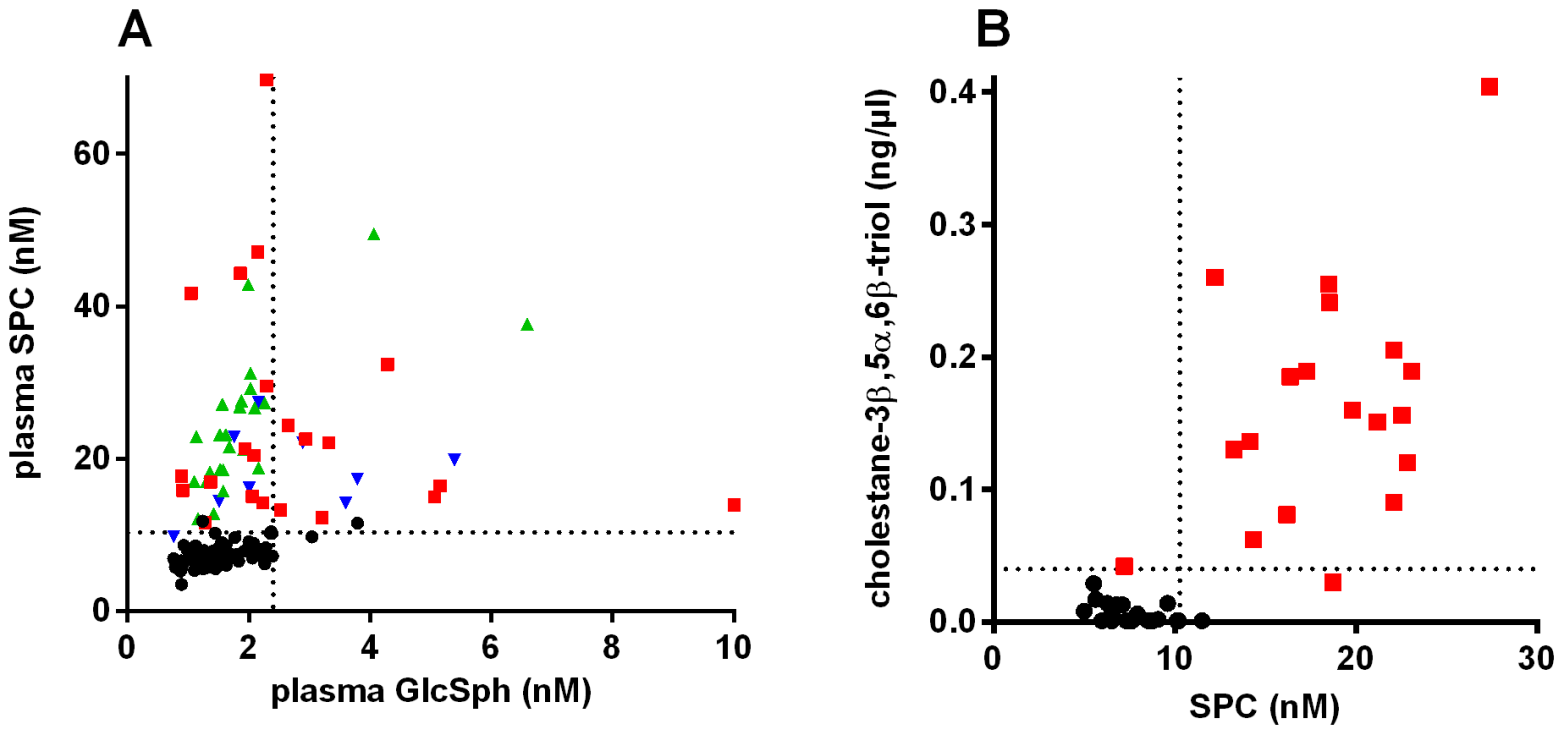
Biomarker correlation plots A: SPC and GlcSph. The control subjects are black circles, the NP-C patients are colored by miglustat treatment status as for Figure 3E. A Spearman correlation analysis gave (r, p value) for control (0.48, <0.0001), NP-C miglustat naïve (−0.11, 0.63), NP-C miglustat treated (0.75, <0.0001) and miglustat unknown (0.28, 0.46). The dotted horizontal and vertical lines are the 95% percentile of the entire control group for SPC (10.3 nM) and GlcSph (2.4 nM) respectively. **B: SPC and cholestan-3β,5α,6β-triol.** For controls (black circles) and NP-C (red squares). The 2 markers did not correlate for the NP-C patients (Spearman r = 0.265, p = 0.273). The horizontal dotted line represents the 95 percentile of normal for cholestan-3β,5α,6β-triol (0.04 ng/µL). The vertical cut-off is 95 percentile of normal for SPC. Values below the assay LOQ were set at the LOQ for SPC (5 nM) and cholestan-3β,5α,6β-triol (0.001 ng/uL).

A correlation plot of SPC and GlcSph indicated that the two markers significantly correlated in controls, but not in NP-C patients (Figure 5A). The NP-C patients with high GlcSph, included 5 miglustat-naïve patients with relatively low SPC (<17 nM).

For 19 controls and 18 NP-C patients the performance of SPC was compared to that of cholestan-3β,5α,6β-triol (Figure 5B). The 2 markers did not correlate for the NP-C patients (Spearman r = 0.156, p = 0.54) suggesting that a combination of the two markers could be the most powerful for diagnosis.

For 32 NP-C patients serial samples were available from follow-up visits (Figure S4 in File S1). SPC in particular was found to be relatively stable with time in the majority of patients. No strong miglustat treatment effect on either biomarker could be deduced from the data.

### Glucosylsphingosine

**Figure 6:**
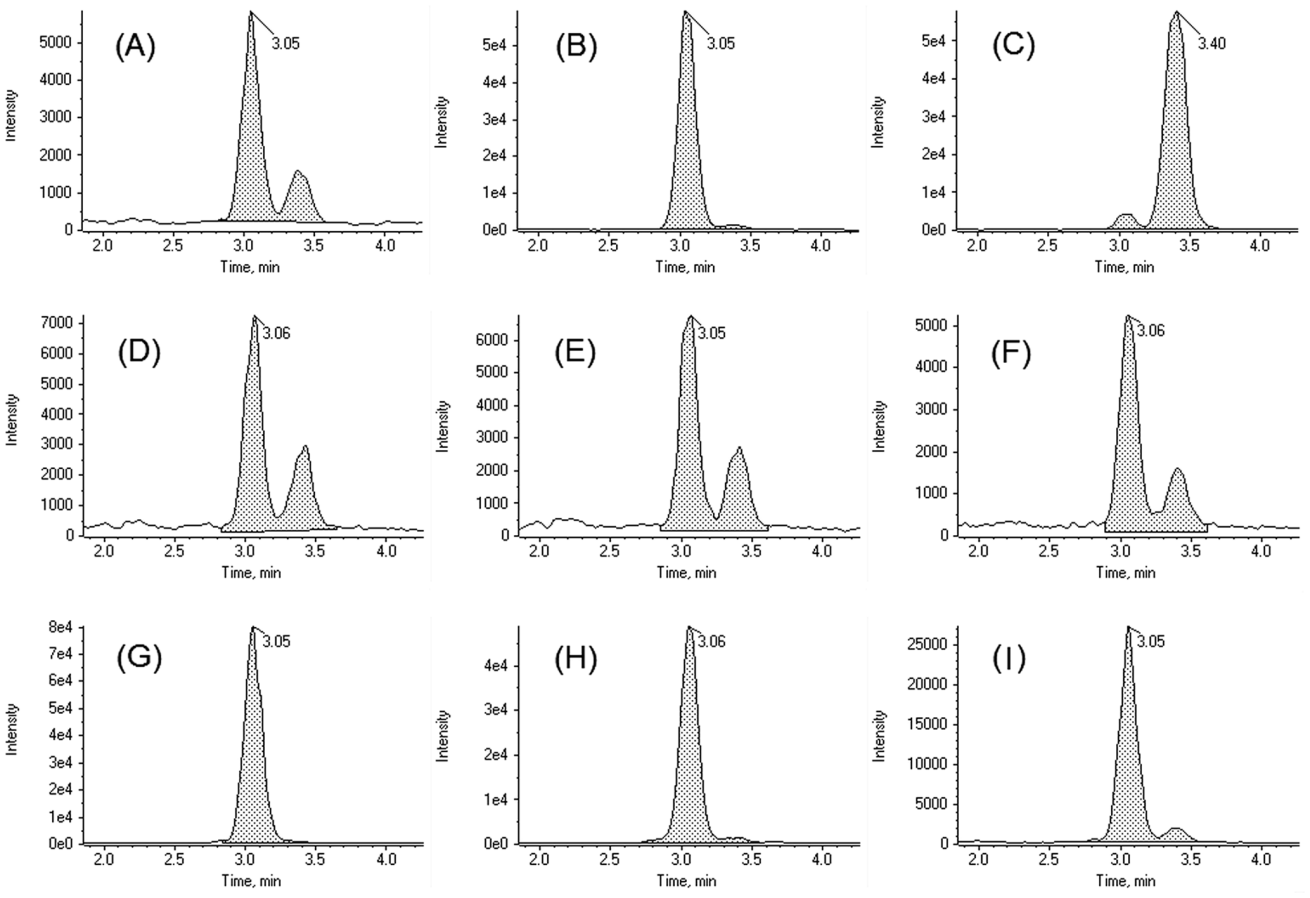
Glucosylsphingosine not galactosylsphingosine is increased in the plasma of NP-C patients. LC-MS/MS chromatograms showing separation of glucosyl- and galactosyl-sphingosine with HILIC chromatography. (A) QC2; (B) QC2 spiked with 5 nM glucosylsphingosine; (C) QC2 spiked with 5 nM galactosylsphingosine; (D) control sample 1; (E) control sample 2; (F) control sample 3; (G) NP-C sample 1; (H) NP-C sample 2; (I) NP-C sample 3.

Subsequent to the main study a sub-study was designed to investigate if the hexosylsphingosine peak corresponded to glucosylsphingosine (GlcSph) or galactosylsphingosine (GalSph). To achieve separation of GlcSph and GalSph it was necessary to switch to a HILIC stationary phase for the chromatography so that interactions were dominated by the polar sugar moiety. GlcSph was found to elute before GalSph (Figure 6). In the control samples there was ∼3-fold more GlcSph than GalSph. In the three NP-C patient samples, the increase above normal levels was dominated by GlcSph, leading to an increase in the GlcSph/GalSph ratio (to >12) (Figure 6).

## Discussion

NP-C is a devastating neurovisceral disease in which the time from neurological symptom onset to diagnosis is still too long and it must be feared that a number of cases remain undiagnosed. Biomarkers such as SPC described in this manuscript have the potential to facilitate diagnosis as they are reliable and easy to use with large numbers of samples.

For clinical qualification of biomarkers it is essential to validate the assay to establish overall bioanalytical precision, accuracy and robustness, as well as to identify potential pitfalls. The assay has excellent precision and good accuracy and it is easy to run in 96-well format, making it appropriate for moderate through-put screening. The markers showed good stability in the conditions tested including for 4 days in plasma at 4°C and for 5 h in blood at room temperature. Additionally, the biomarker levels were unaffected by the anti-coagulant used in the blood draw. After a period of ∼80 days storage at −20°C an upward drift of SPC in the low QC sample was observed. This is at odds with the rest of the stability data, as levels in samples stored at −80°C for similar lengths of time remained stable. Additionally, control samples stored at −80°C for 2 years were in the same range as those taken 1–2 months before measurement.

LC-MS/MS assays are usually developed in individual laboratories and there is a wide range of different instruments and configurations available. It was found that sample measurement could be transferred rapidly across three different instrument platforms, with the same final measured concentrations, despite differences in machine response.

With the validated assay in hand plasma SPC and GlcSph were assessed in a cohort of NP-C patients. Plasma SPC was elevated in NP-C patients independent of age in the range 0–50 years, and of treatment with miglustat. Although limited by the sample set, the ROC clearly demonstrated the ability of SPC to sensitively identify NP-C patients as observed previously in the ZOOM study [4]. SPC was also seen to be elevated in NP-C patient plasma samples in a patent application by Rolfs and Mascher (PCT/EP2012/004756) which became public while completing the work described here.

For GlcSph the increase in plasma levels above normal was present in 41% of miglustat naïve NP-C patients. As miglustat is a glucosylceramide synthase inhibitor it might be expected to lower GlcSph and the data seem to suggest this might be the case, as in the miglustat treated sub-group only 8% of patients had elevated GlcSph, although statistical significance is not reached. GlcSph and SPC did not correlate for miglustat-naïve NP-C patients, indicating that there may be a benefit to maintain the two-analyte assay for NP-C diagnosis.

Plasma lysosphingolipids most probably represent accumulated N-acetylated sphingolipids in the organs (sphingolipidosis), making them complementary to oxysterols as oxidative stress biomarkers for NP-C. The visceral NP-C symptoms of splenomegaly, hepatomegaly and cholestatic jaundice are all heterogeneous, and at least partially age dependent in their presentation [2]. The observation that plasma SPC and GlcSph increases are largely independent of age therefore implies that they are not linked to any one specific visceral symptom.

Although Niemann-Pick diseases type A (NP-A), B (NP-B) and C have different etiologies they exhibit certain clinical, morphological and biochemical similarities including the accumulation of sphingomyelin in the liver and spleen [1], [25]. The recent observation that the 7-ketocholesterol oxysterol marker is also elevated in NP-A and NP-B, both characterized by acid sphingomyelinase deficiency, serves to support the link between the sphingomyelin/SPC and cholesterol/oxysterol axes [26]. In fact, there is a rich base of literature demonstrating an interaction between sphingomyelin and cholesterol both on the physical chemical level within membranes and through regulating one another's synthesis [27].

The reported magnitude of glucosylceramide changes in peripheral organs of NP-C patients varies between a factor of 2- and 20- [17], [28], a fact that may be reflected in the observed heterogeneity of increases in plasma GlcSph seen here. Fan et al recently published an extensive targeted analysis of N-acetylated sphingolipids in the plasma of NP-C patients [16]. Increases in monohexosylceramides were among the most marked changes, and were reportedly augmented by miglustat therapy. The latter observation is not confirmed by the GlcSph data reported here, suggesting that glucosylceramide and GlcSph are not necessarily correlated.

There is already strong evidence that GlcSph is markedly elevated in the plasma of Gaucher patients [12], [29], with the increase being much larger than that seen here for NP-C. Similarly, SPC was recently observed to be elevated in blood spots from patients with NP-B [15]. The fact that the assay described here will likely be of use for several LSDs offers a potential cost saving benefit. The possibility to use dried blood spots could be particularly attractive for physicians far from tertiary centers. Additionally, due to the rarity of LSDs, physicians often find it difficult to diagnose patients and screening for multiple diseases offers the chance to serendipitously identify patients who might otherwise be missed.

The assay for SPC has appropriate through-put and sensitivity that it could both replace the filipin test in the NP-C diagnostic algorithm [30] and be used to identify NP-C patients in pre-specified populations with a prevalence of above 1%, providing confirmatory genetic testing is utilized. Pre-specified populations with sufficient suspicion of NP-C would include infants with neonatal cholestatic liver disease [2], patients with hepatosplenomegaly [31], the intellectually disabled [32] and adults with neurological and psychiatric symptoms [4]. Together with differential clinical diagnosis, the standard enzymatic tests for Gaucher and NP-A/B could also be used as an alternative to sequencing to differentiate these disorders from NP-C in patients with elevated plasma SPC and GlcSph. However, based on the available data [12], [29] it looks quite possible that future studies will establish that Gaucher and NP-A/B can be differentiated from NP-C based on plasma levels of GlcSph and SPC respectively.

The LC-MS/MS assay described here for the measurement of the lysosphingolipids SPC and GlcSph in human plasma is precise, accurate, robust, stable to differences in sampling conditions and simple to run at moderate through-put. These factors should enable clinical implementation. As these markers are relevant to other LSDs, the assay validation data will be of more general use to clinical scientists and laboratories. SPC is confirmed as being elevated in the plasma of NP-C patients and the sensitivity/specificity of 100%/97% in the studied population is highly suggestive of utility in the diagnosis of NP-C, where it could help identify patients for confirmatory genetic testing. Median plasma GlcSph was elevated 1.6-fold in the miglustat-naïve NP-C patients, and did not correlate with SPC. Inclusion of GlcSph measurement with SPC in the assay may improve sensitivity for borderline cases. Further, these two markers may eventually enable tracking of treatment effects on the sphingolipidosis observed in NP-C and will provide a powerful complement to the recently identified oxysterol markers [9], [10].

## Supporting Information

File S1 **Supplemental tables and figures.**
(DOCX)
