# Supplementary material for: Plasma Lysosphingomyelin Demonstrates Great Potential as a Diagnostic Biomarker for Niemann-Pick Disease Type C in a Retrospective Study
Source: PLoS One. 2014 Dec 5;9(12):e114669. doi: 10.1371/journal.pone.0114669 (PMC4257710; doi:10.1371/journal.pone.0114669)
Supplement: File S1 — Supplemental tables and figures. (DOCX) [file pone.0114669.s001.docx]

**Plasma lysosphingomyelin demonstrates great potential as a diagnostic biomarker for Niemann-Pick disease type C in a retrospective study**

Richard W. D. Welford*^a^*, Marco Garzotti*^a^*, Charles Marques Lourenço^b^, Eugen Mengel^c^, Thorsten Marquardt^d^, Janine Reunert^d^, Yasmina Amraoui^c^, Stefan A. Kolb*^a^*, Olivier Morand*^a^*, Peter Groenen*^a^*

**Supplementary Material**

**Supplemental Tables**

| **Analyte** | **Analyte MF** | **ISTD MF** | **ISTD normalized MF** | **CV of ISTD normalized MF** |
| --- | --- | --- | --- | --- |
| SPC low | 1.00±0.05 | 0.96±0.06 | 1.05±0.07 | 6.23 |
| SPC high | 0.97±0.06 | 0.97±0.05 | 1.00±0.01 | 1.04 |
| GlcSph low | 1.06±0.07 | 0.92±0.02 | 1.16±0.09 | 7.98 |
| GlcSph high | 0.98±0.06 | 0.95±0.04 | 1.03±0.09 | 8.34 |

**Table S1:** Matrix factors (MF). Following extraction plasma samples from 6 different donors were reconstituted with high, low or no analytes. Data are reported +/- standard deviation.

| **Batch** | **Value** | **SPC QC2** | **SPC QC3** | **SPC QC4** | **GlcSph QC2** | **GlcSph QC3** | **GlcSph QC4** |
| --- | --- | --- | --- | --- | --- | --- | --- |
| QTRAP4000 | mean concentration (nM) | 9.71 | 101.85 | 374.43 | 0.65 | 13.67 | 46.12 |
|  | CV [%] | 12.2 | 3.2 | 1.6 | 9.0 | 3.4 | 5.5 |
| QTRAP5500 | mean concentration (nM) | 8.88 | 106.21 | 382.37 | 0.56 | 12.11 | 41.98 |
|  | CV [%] | 20.1 | 0.6 | 5.7 | 4.9 | 1.8 | 4.5 |
| QTRAP6500 | mean concentration (nM) | 9.7 | 98.42 | 379.98 | 0.63 | 11.84 | 39.09 |
|  | CV [%] | 20.1 | 0.6 | 5.7 | 14.0 | 8.7 | 5.3 |

**Table S2:** Concentration values of SPC and GlcSph determined in QC samples on different instrument platforms. The QTRAP6500 was the standard platform used for the majority of the studies

| **SPC** | **control**  **(n = 70)** | **NP-C**  **(n =57)** | **NP-C miglustat naive**  **(n = 22)** | **NP-C miglustat treated**  **(n = 25)** | **NP-C miglustat status unknown**  **(n = 9)** |
| --- | --- | --- | --- | --- | --- |
| **Number of values** | 70 | 57 | 22 | 25 | 9 |
|  |  |  |  |  |  |
| **Minimum** | 3.46 | 7.23 | 11.57 | 12.09 | 9.77 |
| **25% Percentile** | 6.48 | 15.79 | 14.78 | 17.86 | 14.27 |
| **Median** | 7.27 | 20.41 | 19.04 | 22.88 | 17.31 |
| **75% Percentile** | 8.24 | 27.21 | 30.22 | 27.45 | 22.47 |
| **Maximum** | 11.80 | 69.73 | 69.73 | 49.49 | 27.38 |
|  |  |  |  |  |  |
| **5% Percentile** | 5.37 | 11.39 | 11.67 | 12.28 | 9.77 |
| **95% Percentile** | 10.31 | 47.34 | 66.34 | 47.48 | 27.38 |
|  |  |  |  |  |  |
| **Mean** | 7.46 | 23.02 | 24.43 | 24.15 | 18.22 |
| **Std. Deviation** | 1.53 | 11.30 | 14.56 | 8.97 | 5.37 |
| **Std. Error of Mean** | 0.18 | 1.50 | 3.11 | 1.79 | 1.79 |

| **GlcSph** | **control**  **(n = 70)** | **NP-C**  **(n =57)** | **NP-C miglustat naive**  **(n = 22)** | **NP-C miglustat treated**  **(n = 25)** | **NP-C miglustat status unknown**  **(n = 9)** |
| --- | --- | --- | --- | --- | --- |
| **Number of values** | 70 | 57 | 22 | 25 | 9 |
|  |  |  |  |  |  |
| **Minimum** | 0.76 | 0.76 | 0.89 | 1.10 | 0.76 |
| **25% Percentile** | 1.12 | 1.47 | 1.74 | 1.39 | 1.64 |
| **Median** | 1.40 | 1.99 | 2.26 | 1.62 | 2.16 |
| **75% Percentile** | 1.82 | 2.59 | 3.24 | 2.03 | 3.70 |
| **Maximum** | 3.79 | 10.00 | 10.00 | 6.59 | 5.39 |
|  |  |  |  |  |  |
| **5% Percentile** | 0.84 | 0.91 | 0.89 | 1.11 | 0.76 |
| **95% Percentile** | 2.39 | 5.51 | 9.27 | 5.83 | 5.39 |
|  |  |  |  |  |  |
| **Mean** | 1.51 | 2.38 | 2.80 | 1.95 | 2.65 |
| **Std. Deviation** | 0.56 | 1.59 | 2.00 | 1.13 | 1.42 |
| **Std. Error of Mean** | 0.07 | 0.21 | 0.43 | 0.23 | 0.47 |

**Table S3:** Summary statistics of SPC and GlcSph in the control group, all NP-C patients (1 sample per patient) and the NP-C group split based on miglustat status at the time of sampling.

**Supplemental Figures**


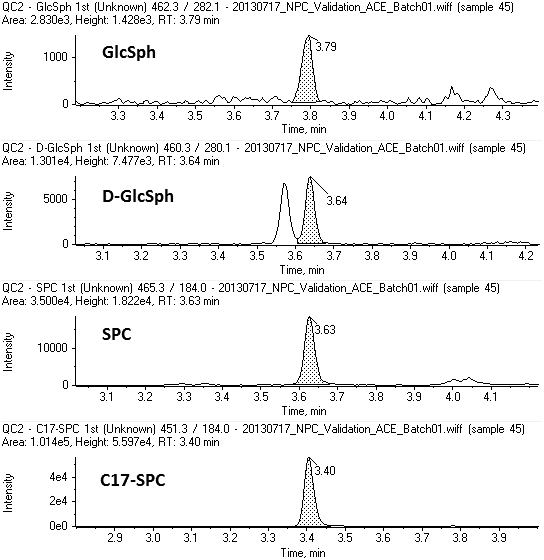


**Figure S1: Chromatographic elution profiles for the two analytes and two ISTDs acquired for a pool of human EDTA-plasma (QC2).** The D-GlcSph ISTD elutes as a split peak, this has been noted elsewhere and is presumed to be a result of *cis*-*trans* isomerization [[1](#_ENREF_1)]. Only the later eluting peak was integrated because it eluted closer to the GlcSph analyte and provided reproducible automated integration.

**Figure S2: Long term stability of measured concentration of plasma QC samples for SPC and GlcSph**. QC2 for SPC (A) and GlcSph (B); QC3 for SPC (C) and GlcSph (D); QC4 for SPC (E) and GlcSph (F). The central horizontal dashed line shows the validation determined average concentration and the outer dotted lines delineate the standard acceptance criteria.

**Figure S3: Stability of** **SPC (A) and GlcSph (B) in fresh EDTA-blood** **SPC (A) and GlcSph (B)** from three donors. EDTA-blood was incubated at room temperature and then converted to plasma at the indicated time points. Subsequently all plasma samples were analyzed in a single batch. Each trace (circles, triangles, squares) represents blood from an individual donor.


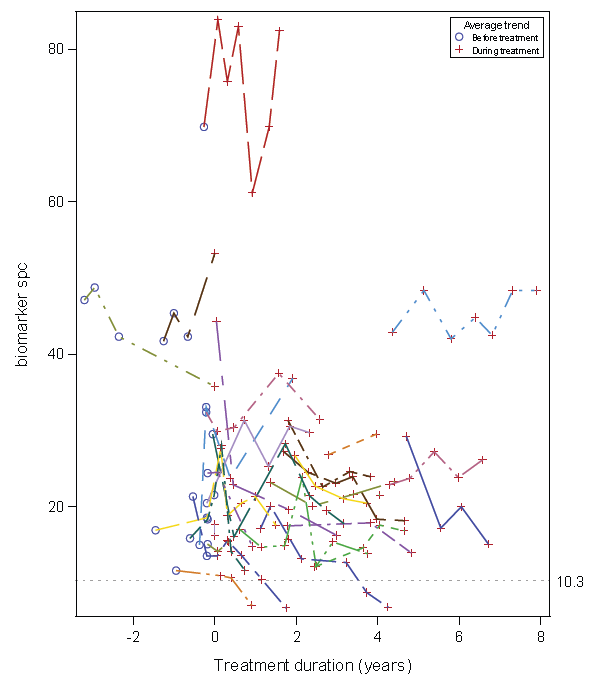


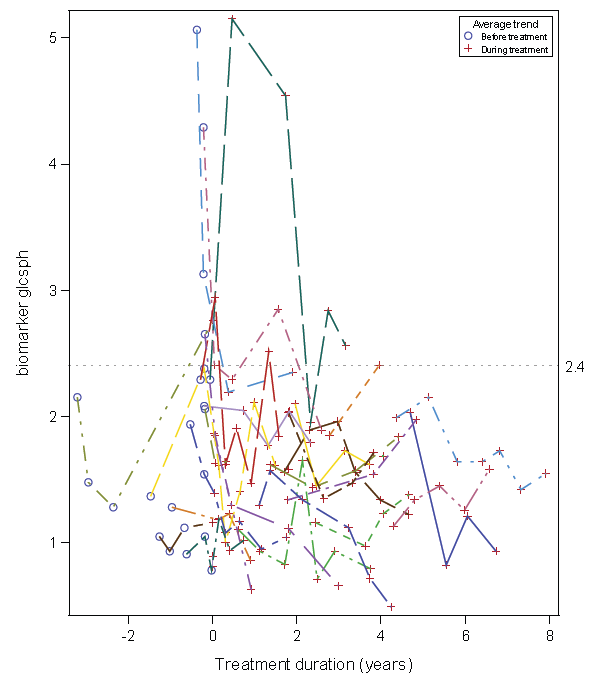


**Figure S4: Plasma SPC and GlcSph in serial samples from NP-C patients**. Data is plotted relative to initiation of treatment with miglustat. Each trace represents an individual patient. The dotted horizontal line at 10.3nM (SPC) and 2.4nM (GlcSph) is the 95 percentile of the control group.

**References**

**1. Boutin M, Gagnon R, Lavoie P, Auray-Blais C (2012) LC-MS/MS analysis of plasma lyso-Gb3 in Fabry disease. Clin Chim Acta 414: 273-280.**
